# Coordination of carbon and nitrogen accumulation and translocation of winter wheat plant to improve grain yield and processing quality

**DOI:** 10.1038/s41598-020-67343-5

**Published:** 2020-06-25

**Authors:** Xin Huang, Chenyang Wang, Junfeng Hou, Chenyang Du, Sujun Liu, Juan Kang, Hongfang Lu, Yingxin Xie, Tiancai Guo, Dongyun Ma

**Affiliations:** 1 College of Agronomy/National Engineering Research Center for Wheat, Henan Agricultural University, Zhengzhou, 450046 China; 2 The National Key Laboratory of Wheat and Maize Crop Science, Henan Agricultural University, Zhengzhou, 450046 China

**Keywords:** Plant sciences, Plant development, Plant physiology, Plant reproduction

## Abstract

The objective of this work was to characterize the accumulation of carbon (C) and nitrogen (N), and the translocation of wheat (*Triticum aestivum* L.) cultivars to achieve both high-quality and high-yield. Twenty-four wheat cultivars, including 12 cultivars containing high-quality gluten subunit 5 + 10 at *Glu-D1*, and 12 cultivars with no *Glu*-*D1* 5 + 10, were planted at Yuanyang and Xuchang in Henan Province, during 2016–2017, and 2017–2018 cropping seasons. Wheat cultivars containing *Glu-D1* 5 + 10 had an advantage in grain quality traits. Significant difference (*P* < 0.05) was observed for grain protein concentration (GPC) between 5 + 10 group and no 5 + 10 group. Grain yield (GY) was significantly correlated with kernel number (KN) (r = 0.778, *P* < 0.01), thousand-kernel weight (TKW) (r = 0.559, *P* < 0.01), dry matter accumulation at post-anthesis (r = 0.443, *P* < 0.05), and stem water-soluble carbohydrate (WSC) accumulation (r = 0.487, *P* < 0.05) and translocation amount (r = 0.490, *P* < 0.05). GPC, dough stability time (DST) and nitrogen agronomic efficiency (NAE) were significantly correlated with nitrogen accumulation (NAA) at maturity stage (r = 0.524, = 0.404, = 0.418, *P* < 0.01, < 0.05, < 0.05, respectively), and nitrogen translocation amount (r = 0.512, = 0.471, = 0.405, *P* < 0.05, < 0.05, < 0.05, respectively). These results suggest that good-quality, high-yield, and high-efficiency could achieve through the selection of high-quality wheat cultivars and coordination of C and N accumulation and translocation. High-quality gluten subunit gene *Glu-D1* 5 + 10 and stem WSC could be used as a selection index for breeding and production of high-quality and high-yield wheat.

## Introduction

Wheat (*Triticum aestivum* L.) is one of the most widely planted crops in the world. Compared to other crops, wheat accounts for the largest planting area, total production and trade volume^1^. As our population continues to grow, an important goal is to increase the overall yield of wheat^2^. On the other hand, the quality of wheat flour utilized for the production of different food products such as bread, noodles, cakes, and biscuits^3–5^, has gained much attention. China, the largest wheat producer and consumer in the world, has a wheat planting area of ~ 24 million ha and an annual production of ~ 115 million Mg^6^. Since the 1980s, there have been extensive efforts to improve the quality of wheat processing, especially for strong gluten wheat^7–9^. However, due to the interconnection between carbon (C) and nitrogen (N) metabolism, some high-quality wheat cultivars are often accompanied by a decline of grain yield (GY). With economic development, increasing population, and eco-environmental problems becoming more and more serious, achieving high-quality, high-yield, and high-efficiency in wheat production are the key goals.

Carbon (C) and N accumulation and transportation are the most critical processes for plant growth and development, and these processes determine the GY and quality^10–12^. It is well known that the wheat grain weight depends on the photosynthates that are transferred directly to the wheat grain and assimilate remobilization from vegetative tissues^13,14^. The photo-assimilation during the grain filling stage is very important for determining the final wheat grain weight^15^, and usually contributes to about 50–67% of total grain dry weight^10^. Water-soluble carbohydrate (WSC) accumulated in the wheat stem and sheath could be redistributed to grain during the later stage of grain filling^16^ and is an important C source for wheat grain and positively correlates to grain yield^17,18^. Moreover, the N in wheat grain comes from both the redistribution of vegetative tissue accumulated N pre-anthesis, and the N assimilated during grain filling^19^. The remobilization of N from the vegetative tissues usually formed the predominant N source and accounted for the grain N content, ranging from 51 to 91%^12,20^. Masoni et al.^14^ reported that the grain carbohydrate coming from the remobilization was lower than 30%, while the grain N coming from the remobilization of N that accumulated pre-anthesis accounted for 73–82%. However, the increase in wheat WSC concentration can reduce the concentration of N in the shoots, and this effect becomes more important as modern breeding techniques result in wheat cultivars with higher WSC content^21^. Thus, it is important to coordinate the C and N accumulation and translocation in order to improve the GY and quality.

The Huang-Huai Winter Wheat Zone (HHWWZ) is the largest wheat producing area in China, and accounts for about 65% of the total national wheat production^6^. Owing to an irrigation area with intensive annual double-crop wheat–maize planting system, the major wheat production objectives in this area are to enhance the cultivar’s yield potential, improve processing quality for bread and noodles, and enhance nitrogen use efficiency (NUE). Many efforts have made to find an evaluation criterion for producing or breeding wheat with high yield, improved grain quality and high NUE by investigating the differences among genotypes with different yield level^22–24^, grain quality^25^, and NUE^26^. The ability to store and remobilize large amounts of WSC to grains was suggested as a selection criterion for wheat breeding^27^. Tian et al.^22^ found that the N assimilation ability prior to anthesis and the N remobilization ability post-anthesis strongly associated with GY and NUE. Currently, many newly-bred high-quality wheat cultivars also exhibit a very high yield potential. However, in practice production, the yield or quality of these cultivars have not been fully demonstrated due to environmental, cultivation management, and various other reasons. Thus, understanding the characteristics of C and N accumulation, translocation and distribution of wheat cultivar with high-yield and high-quality potential, will provide useful information for wheat production. High molecular weight glutenin subunits (HMW-GS), the important composition for gluten, are encoded by the *Glu-A1*, *Glu-B1*, *Glu-D1*, and the allelic variation at these loci play a key role in determining grain quality^28,29^. It has been accepted that *Glu-D1* loci had great contribution to dough strength than *Glu-A1* and *Glu-B1*; At *Glu-D1*, 5 + 10 is significantly better than those of their counterpart allelic variation for Farinograph stability and accepted as wheat high quality (dough strength) subunit^30,31^. In this study, we planted 24 modern wheat cultivars, with high-quality (HMW-GS 5 + 10) and high-yield potential in two growing sites. We investigated the differences in dry matter accumulation and translocation, and N assimilation and remobilization among these cultivars. Our findings can provide useful information for producing and breeding high-quality and high-yield wheat in different wheat cultivation environments.

## Results

As listed in Table 1, analyses of variance were conducted for GY, kernel number (KN), thousand-kernel weight (TKW), plant height (PH), anthesis date (AD), aboveground biomass at jointing stage (AGBM-J), aboveground biomass at anthesis stage (AGBM-A), aboveground biomass at maturity stage (AGBM-M), WSC accumulation amount of stem peduncle (WSC-P), WSC accumulation amount of stem lower internodes (WSC-L), WSC accumulation amount of the total stem (WSC-T), WSC translocation amount of stem peduncle (WSCT-P), WSC translocation amount of stem lower internodes (WSCT-L), WSC translocation amount of the total stem (WSCT-T), leaf area index at jointing stage (LAI-J) leaf area index at anthesis (LAI-A), plant N accumulation at anthesis (NAA), plant N accumulation at maturity (NAM), N translocation amount to grain (NTA), N agricultural efficiency (NAE), grain protein concentration (GPC), WGC (wet gluten content), dough stability time (DST), dough water absorption rate (DWR). Cultivar, environment and cultivar × environment contributed significantly to variation in most of the traits. Significant environment effects were observed for GY, KN and LAI-J. No cultivar × environment effect was exerted on DST.

**Table 1:** Mean square from a combined analysis of variance for yield, processing quality and physiological traits of 24 winter wheat cultivars across four environments.

Source of variation	Cultivar	Environment	Cultivar × environment	Error
df	23	3	68	95
GY	5,712.00	140,667.80**	7,253.19	5,571.93
KN	8.91	449.28**	10.97	8.50
TKW	30.33**	829.08**	11.22**	2.99
PH	45.57**	227.11**	7.60	7.02
AD	9.76**	1,189.84**	5.22**	1.67
AGBM-J	2,014,053.47*	43,347,896.78**	1,331,082.86	1,041,197.37
AGBM-A	3,998,400.27**	1.70E8**	3,881,248.42**	1,336,456.22
AGBM-M	5,496,052.12*	1.38E8**	7,806,619.61*	2,513,435.98
NSC-P10	11,460.58**	689,271.63**	10,827.54**	993.20
NSC-L10	93,535.79**	6,105,397.06**	77,108.63**	13,470.26
NSC-T10	134,394.92**	10,234,925.24**	113,247.34**	21,507.03
NSCT-P	11,714.47**	709,114.14*	10,448.19**	894.57
NSCT-L	89,186.39**	6,265,604.72**	74,492.28**	12,671.09
NSCT-T	129,436.44**	10,521,312.56**	107,713.30**	19,996.28
LAI-J	0.57	23.29**	0.38	0.39
LAI-A	1.21**	50.19**	0.97**	0.34
NAA-A	1,176.59**	19,228.52**	1,379.66**	395.17
NAA-M	2,125.18**	120,462.26**	2,139.99**	636.97
NTM	1,327.59**	185,412.35**	2,747.70**	542.53
GPC	4.45**	52.46*	1.31**	0.27
WGC	64.51**	282.32**	37.53**	1.07
DST	121.57**	309.71**	20.91	23.61
DWR	44.83**	114.48**	3.72**	1.27
NAE	41.05**	1,041.25**	39.22**	18.18

*GY* grain yield, *KN* kernels number, *TKW* thousand-kernel weight, *PH* plant height, *AD* anthesis date, *AGBM-J* aboveground biomass at jointing stage, *AGBM-A* aboveground biomass at anthesis stage, *AGBM-M* aboveground biomass at maturity, *WSC-P10* WSC accumulation amount of peduncle at 10 days post-anthesis, *WSC-L10* WSC accumulation amount of lower internodes at 10 days post-anthesis, *WSC-T10* total stem WSC accumulation amount at 10 days post-anthesis, *WSCT-P* WSC translocation amount of peduncle, *WSCT-L* WSC translocation amount of lower internodes, *WSCT-T* total stem WSC translocation amount, *LAI-J* leaf area index at jointing stage, *LAI-A* leaf area index at anthesis, *NAA-A* nitrogen accumulation amount at anthesis, *NAA-M* nitrogen accumulation amount at maturity, *NTA* nitrogen translocation amount, *GPC* grain protein concentration, *WGC* wet gluten content, *DST* dough stability time, *DWR* dough water absorption rate, *NAE* nitrogen agricultural efficiency.

*Significant at the 0.05 probability level.

**Significant at the 0.01 probability level.

### Grain yield, yield component, and flour quality parameters

As presented in Table 2, large differences were observed for GY among wheat cultivar ‘Bainong 307’, ‘Tianmin 198’, ‘Yunong 186’, ‘Pingan 11’, and ‘Zhoumai 32’, with the corresponding yield of 6,185.17, 6,182.76, 7,708.39, 7,950.02 and 7,690.08 kg ha^−1^, respectively. Cultivar ‘Pingan 11’ and ‘Zhoumai 27’ got the highest KN (21.89 10^3^ m^-2^ and 21.45 10^3^ m^-2^, respectively), whereas ‘Xinhuamai 818’ and ‘Shangmai 156’ got the lowest KN. Big differences were also observed for TKW between ‘Zhengmai 119’, ‘Xinhuamai 818’, ‘Fengdecun 5’ and most other cultivars. Cultivar ‘Zhengmai 119’ had the highest PH (79.0 cm), whereas ‘Tianmin198’ got the lowest PH (67.0 cm). The GPC ranged from 13.23 g 100 g^−1^ for ‘Pingan11’ to 16.28 g 100 g^−1^ for ‘Xinmai26’. Cultivar ‘Zhengmai 119’ got the highest WGC of 35.62 g 100 g^−1^, whereas ‘Zhoumai 27’ got the lowest value of 20.99 g 100 g^−1^. The DST ranged from 19.12 min for ‘Xinmai 26’ to 1.86 min for ‘Luohan 19’. Cultivar ‘Zhengmai 369’ got the highest DWR with the value being 66.57 ml 100 g^−1^, whereas ‘Tianmin 198’ got the lowest value being 55.00 ml 100 g^−1^. Considering GY and quality traits, cultivar ‘Zhengmai 119’, ‘Zhengmai 369’, ‘Zhoumai 32’, and ‘Fengdecun 5’ have the potential of high-quality and high-yield. No significant difference was observed for most of the quality traits (except for GPC), between cultivars containing HMW-GS 5 + 10 and those without 5 + 10. However, 83.33% (10/12) of the cultivars with 5 + 10 had DST > 7.0 min, but only 58.3% (7/12) of the cultivars without 5 + 10 had DST of more than 7.0 min. These results suggest that HMW-GS 5 + 10 are still an important criterion for improving grain quality.

**Table 2:** Yield components, morphological and processing quality traits of 24 wheat cultivars averaged for four environments.

HMW-GS 5 + 10	Cultivar	Traits								
		GY (kg ha^−1^)	KN (10^3^ m^−2^)	TKW(g)	PH (cm)	AD (days)	GPC (%)	WGC (%)	DST (min)	DWR (%)
With	Xinmai 26	6,764.24	18.31	43.45	73.93	175	16.28	29.15	19.12	63.98
	Zhengmai 366	6,698.66	19.34	40.73	73.00	174	15.66	33.23	13.68	63.02
	Zhengmai 119	7,650.00	19.69	45.71	79.00	174	15.64	35.62	13.80	63.14
	Zhengmai 369	7,558.05	20.48	43.39	76.23	174	14.83	32.21	12.13	66.57
	Zhengmai 7698	7,059.17	19.44	42.70	71.83	176	15.01	31.46	10.68	61.45
	Fengdecun 5	7,626.14	19.89	45.09	70.28	174	15.07	31.13	12.73	60.00
	Luomai 31	6,856.77	18.20	44.29	76.28	177	14.80	35.49	5.73	60.97
	Zhoumai 36	7,370.74	19.92	43.50	75.73	177	15.26	28.56	16.80	55.53
	Zhoumai 33	6,677.78	19.36	40.57	74.68	176	15.41	32.25	14.67	59.88
	Yumai 158	7,435.45	19.68	44.44	76.90	177	14.66	23.81	9.34	57.66
	Bainong 207	7,184.73	19.49	43.34	76.43	177	14.48	33.64	5.10	57.70
	Bainong 307	6,185.17	18.13	40.11	67.08	176	13.60	27.99	7.95	57.55
	Mean	*7,088.91A*	*19.32 A*	*43.11A*	*74.94A*	*176 A*	*15.06 A*	*31.21 A*	*11.81 A*	*60.54 A*
Without	Yunong 186	7,708.39	20.37	44.49	68.83	176	13.84	31.13	2.73	55.73
	Tianmin 198	6,182.76	19.03	38.20	67.00	174	13.62	26.95	2.03	55.00
	Bainong 4199	7,196.31	19.72	42.92	70.00	175	13.88	29.46	15.77	59.17
	Pingan 11	7,950.02	21.89	42.71	71.43	175	13.23	24.48	8.28	58.78
	Luomai 26	7,468.46	20.55	42.74	72.32	179	13.71	29.94	5.68	59.04
	Zhoumai 27	7,337.81	21.45	40.22	73.68	176	13.58	20.99	7.10	59.14
	Zhoumai 32	7,690.08	21.12	42.82	74.78	176	15.84	33.19	17.05	59.92
	Xinhuamai 818	6,477.75	16.84	45.22	77.58	177	15.57	34.47	7.12	57.37
	Xinong 979	7,341.34	20.82	41.47	76.73	175	14.96	30.46	16.58	64.73
	Saidemai 1	7,513.65	19.81	44.60	75.40	176	14.66	32.50	9.03	59.50
	Shangmai 156	6,480.05	17.89	42.68	76.83	173	13.25	30.31	2.33	58.58
	Luohan 19	6,524.76	19.53	39.28	75.28	176	13.73	32.62	1.86	61.70
	Mean	*7,155.94 A*	*19.92 A*	*42.28 A*	*73.32 A*	*176 A*	*14.15 B*	*29.71 A*	*7.96 A*	*59.06 A*

The italicized data followed by different capital letters in the same column indicate a significant difference at *P* < 0.05.

*GY* grain yield, *KN* kernels number, *TKW* thousand-kernel weight, *PH* plant height, *AD* anthesis date (from seedling emergence to anthesis), *GPC* grain protein concentration, *WGC* wet gluten content, *DST* dough stability time, *DWR* dough water absorption rate.

### Physiological traits and nitrogen efficiency

There were great differences for AGBM, WSC, and LAI among these wheat cultivars (Table 3). Just for AGBM-J and AGBM-A, cultivar ‘Sandemai 1’ showed a higher value than ‘Bainong 307’. The WSC-T ranged from 1,458.74 kg ha^−1^ for ‘Sandemai 1’ to 963.26 kg ha^−1^ for ‘Tianmin 198’. Large differences for WSCT-P occurred among ‘Xinong 979’, ‘Zhoumai 32’, ‘Tianmin 198’, ‘Xinmai 26’, ‘Zhengmai 366’, ‘Fengdecun 5’, and ‘Zhengmai 119’. For WSCT-L and WSCT-T, cultivar ‘Saidemai 1’ got the highest value of 1,133.88 kg ha^−1^ and 1,395.82 kg ha^−1^, respectively; whereas ‘Tianmin 198’ got the lowest one, with the amount being 752.18, and 920.17 kg ha^−1^, respectively. Wheat cultivar ‘Luomai 31’ got the highest LAI at jointing stage (4.41), whereas ‘Luomai 26’ and ‘Pingan 11’ got the highest LAI at anthesis with the value being 6.20 and 5.80, respectively.

**Table 3:** Physiological traits of 24 wheat cultivars averaged for four environments.

HMW-GS 5 + 10	Cultivar	Traits							
		AGBM-J	AGBM-A	WSC-T	WSCT-P	WSCT-L	WSCT-T	LAI-J	LAI-A
With	Xinmai 26	5,101.60	10,115.72	1,070.08	112.16	922.77	1,034.93	3.33	4.96
	Zhengmai 366	5,918.84	12,208.88	1,044.35	145.71	867.86	1,013.57	3.90	5.37
	Zhengmai 119	5,915.08	12,139.94	1,148.10	187.71	923.93	1,111.64	3.89	5.42
	Zhengmai 369	6,132.47	10,664.31	1,093.77	237.03	814.72	1,051.75	3.74	4.71
	Zhengmai 7698	6,857.84	10,616.00	1,311.58	246.08	1,024.82	1,270.91	4.01	5.26
	Fengdecun 5	5,335.44	10,906.92	1,040.39	191.30	822.74	1,014.04	3.95	5.10
	Luomai 31	5,873.72	11,838.40	1,310.84	219.51	1,047.52	1,267.03	4.41	5.58
	Zhoumai 36	5,699.60	11,027.85	1,341.07	274.27	1,013.82	1,288.09	3.54	5.65
	Zhoumai 33	6,030.93	10,712.76	1,217.48	228.00	960.00	1,188.00	4.00	5.40
	Yumai 158	5,925.85	10,723.60	1,409.30	263.49	1,109.81	1,373.31	3.92	4.76
	Bainong 207	5,161.81	11,050.39	1,331.79	272.82	1,026.44	1,299.26	3.78	4.40
	Bainong 307	5,206.33	9,005.00	1,072.42	215.20	827.41	1,042.61	3.52	4.00
	Mean	*5,763.29 A*	*10,917.48 A*	*1,199.26 A*	*216.11 A*	*946.82 A*	*1,162.93 A*	*3.83 A*	*5.05 A*
Without	Yunong 186	5,237.34	11,445.50	1,357.83	242.35	1,061.14	1,303.48	3.93	5.21
	Tianmin 198	4,414.00	11,085.20	963.26	167.99	752.18	920.17	3.45	4.27
	Bainong 4199	6,437.00	10,305.00	1,216.23	243.08	928.36	1,171.44	3.67	5.69
	Pingan 11	6,542.00	11,401.86	1,329.19	260.35	1,028.83	1,289.17	4.21	5.80
	Luomai 26	5,735.96	10,708.08	1,364.33	279.39	1,056.38	1,335.77	4.33	6.20
	Zhoumai 27	6,412.59	12,725.40	1,350.46	252.73	1,048.58	1,301.32	4.26	5.35
	Zhoumai 32	5,622.25	11,110.42	1,366.26	298.50	1,030.56	1,329.05	4.36	5.50
	Xinhuamai 818	4,746.00	10,273.00	1,261.57	210.77	1,013.27	1,224.04	3.86	5.21
	Xinong 979	5,961.55	10,992.93	1,195.68	304.34	875.25	1,179.59	3.71	5.78
	Saidemai 1	6,953.99	12,241.26	1,458.74	261.94	1,133.88	1,395.82	3.97	5.13
	Shangmai 156	5,621.00	11,449.41	1,276.93	230.44	1,007.63	1,238.07	4.04	5.60
	Luohan 19	6,050.33	9,880.71	1,160.00	223.82	889.86	1,113.68	3.40	5.19
	Mean	*5,811.17 A*	*11,134.90 A*	*1,275.04 A*	*247.98 A*	*985.49 A*	*1,233.47 A*	*3.93 A*	*5.41 A*

The underlined data followed by different capital letters in the same column indicate a significant difference at *P* < 0.05.

*AGBM-J* aboveground biomass at jointing stage, *AGBM-A* aboveground biomass at anthesis stage, *WSC-T* WSC accumulation amount of stem at 10 days post-anthesis, *WSCT-P* WSC translocation amount of peduncle, *WSCT-L* WSC translocation amount of lower internodes, *WSCT-T* WSC translocation amount of total stem, *LAI-J* leaf area index at jointing stage, *LAI-A* leaf area index at anthesis.

As listed in Table 4, great differences were observed among cultivars for N accumulation, translocation and NUE. NAA-A ranged from 158.16 kg ha^−1^ for ‘Luomai 31’ to 222.26 kg ha^−1^ for ‘Zhengmai 7698’. Wheat cultivar ‘Xinmai 26’, ‘Zhengmai 7698’ and ‘Zhoumai 32’ had a higher NAA-M than ‘Bainong 307’, ‘Zhengmai 369’, Zhoumai 27’, ‘Luohan 19’, and ‘Fengdecun 5’. NTA ranged from 144.65 kg ha^−1^ for ‘Bainong 207’ to 206.45 kg ha^−1^ for ‘Zhengmai 366’. Big differences were observed for NAE among ‘Fengdecun 5’, ‘Bainong 307’, ‘Tianmin 198’ and ‘Zhoumai 32’. Cultivar ‘Zhoumai 32’ and ‘Fengdecun 5’ got the highest NAE with the value being 16.34 kg kg^−1^ and 16.27 kg kg^−1^, respectively. No significant difference for carbohydrate accumulation related traits (Table 3), N accumulation and translocation were observed between cultivars containing HMW-GS 5 + 10 and those without 5 + 10. However, there was a tendency that cultivars containing 5 + 10 showed a high N accumulation and translocation, whereas cultivars without 5 + 10 seem had high AGBM, WSC accumulation and translocation.

**Table 4:** Nitrogen accumulation, translocation amount and nitrogen use efficiency of 24 wheat cultivars averaged for four environments.

HMW-GS 5 + 10	Cultivar	Traits				
		NAA-A	NAA-M	NTA	NAE	NUtE
With	Xinmai 26	213.78	277.28	189.26	11.07	27.21
	Zhengmai 366	217.46	264.89	206.45	12.34	26.12
	Zhengmai 119	197.59	234.68	195.97	13.87	34.25
	Zhengmai 369	186.42	216.74	164.15	12.56	35.83
	Zhengmai 7698	222.26	275.16	185.19	14.58	27.39
	Fengdecun 5	203.72	217.01	191.73	16.27	37.16
	Luomai 31	158.16	267.38	151.47	13.51	26.90
	Zhoumai 36	211.33	267.53	184.71	14.69	28.51
	Zhoumai 33	197.37	225.82	189.98	15.61	24.44
	Yumai 158	202.01	243.50	192.47	13.23	31.63
	Bainong 207	187.67	239.08	144.65	11.01	31.20
	Bainong 307	177.84	212.73	145.42	6.86	29.44
	Mean	*197.97 A*	*245.15 A*	*178.45 A*	*12.97 A*	*30.01 A*
Without	Yunong 186	198.75	234.46	180.93	11.09	34.40
	Tianmin 198	184.39	223.53	155.66	6.71	28.72
	Bainong 4199	198.26	234.02	169.63	11.62	32.55
	Pingan 11	190.68	245.03	160.16	14.08	32.83
	Luomai 26	183.73	244.23	155.51	15.29	31.61
	Zhoumai 27	192.83	203.09	200.07	11.61	36.35
	Zhoumai 32	200.20	280.66	162.73	16.34	28.67
	Xinhuamai 818	195.46	247.32	174.54	12.39	26.66
	Xinong 979	178.88	228.01	164.07	13.07	33.27
	Saidemai 1	168.85	248.83	172.77	13.86	33.01
	Shangmai 156	175.49	247.40	149.76	8.47	28.75
	Luohan 19	162.80	177.92	160.51	8.72	35.32
	Mean	185.86 A	234.54 A	167.20 A	11.94 A	*31.85 A*

The italicized data followed by different capital letters indicate a significant difference at *P* < 0.05.

*NAA-A* nitrogen accumulation amount at anthesis, *NAA-M* nitrogen accumulation amount at maturity, *NTA* nitrogen translocation amount, *NAE* nitrogen agricultural efficiency, *NUtE* N utilization efficiency.

### Correlations among grain yield and physiological traits

**Figure 1:**
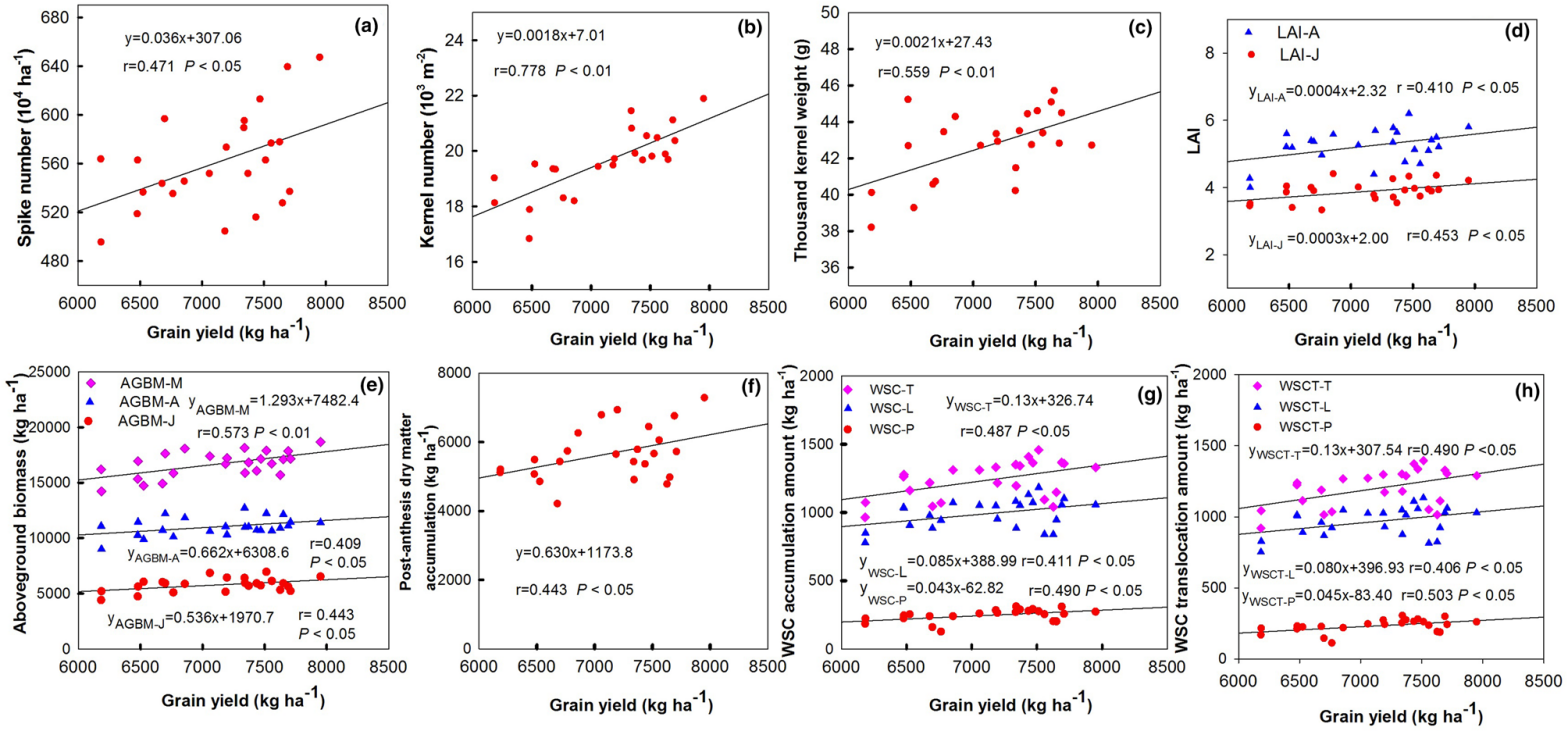
Relationship between grain yield, spike number (**a**), and kernel number (**b**), and thousand kernel weight (**c**), and LAI (**d**), and above ground biomass (**e**), and post-anthesis dry matter accumulation (**f**), and WSC accumulation (**g**), and WSC translocation. *LAI-J and LAI-A* leaf area index at joining stage and anthesis stage respectively; *AGBM-J, AGBM-A, and AGBM-M* above ground biomass at joining stage, anthesis stage, maturity stage respectively; *WSC* water soluble carbohydrate, *WSC-P, WSC-L, and WSC-T* WSC accumulation amount of peduncle, lower internodes, and the total stem, respectively; *WSCT-P, WSCT-L, WSCT-T* WSC translocation amount of peduncle, lower internodes, and the total stem, respectively.

As shown in Figure 1, GY was significantly and positively correlated with SN (r = 0.471, *P* < 0.05), KN (r = 0.778, *P* < 0.01) and TKW (r = 0.559, *P* < 0.01) (Figure 1a, b, c), and the highest correlation was showed between KN and GY. Also, GY was significantly correlated with LAI at jointing stage (r = 0.453, *P* < 0.05), with LAI at anthesis (r = 0.410, *P* < 0.05) (Figure 1d). The plant biomass at each growth stage significantly correlated to GY (Figure 1e), but the highest correlation coefficient was obtained at maturity (r = 0.573, *P* < 0.01) and the lowest correlation appeared at the anthesis stage (r = 0.409, *P* < 0.05). The assimilation amount post-anthesis also positive correlated to GY (r = 0.443, *P* < 0.05) (Figure 1f). In addition, WSC accumulation in peduncle, lower internodes, and the total stem significantly correlated to GY with r = 0.490 (*P* < 0.05), = 0.411 (*P* < 0.05), and = 0.487 (*P* < 0.05), respectively (Figure 1g). WSC translocation in peduncle, lower internodes, and the total stem significantly correlated to GY with r = 0.503 (*P* < 0.05), = 0.406 (*P* < 0.05), and = 0.490 (*P* < 0.05), respectively (Figure 1h). These results indicate increasing dry matter accumulation, especially stem WSC accumulation and translocation would improve GY. Also, the correlation coefficients between GY and physiological traits of wheat cultivars within the same *Glu-D1* allele were also analyzed (Table S1). GY was significantly positively correlated with both KN (r = 0.801, *P* < 0.01) and TKW (r = 0.831, *P* < 0.01) within *Glu-D1* 5 + 10 wheat cultivars, while GY was only significantly correlated with KN (r = 0.806, *P* < 0.01) within no *Glu-D1* 5 + 10 wheat group. GY was positively correlated with WSC-T, WSCT-P, WSCT-L, and WSCT-T regardless of *Glu-D1* allele, but the correlation coefficients were only significant within no *Glu-D1* 5 + 10 wheat group.

### Correlations among grain quality, NAE and physiological traits

**Figure 2:**
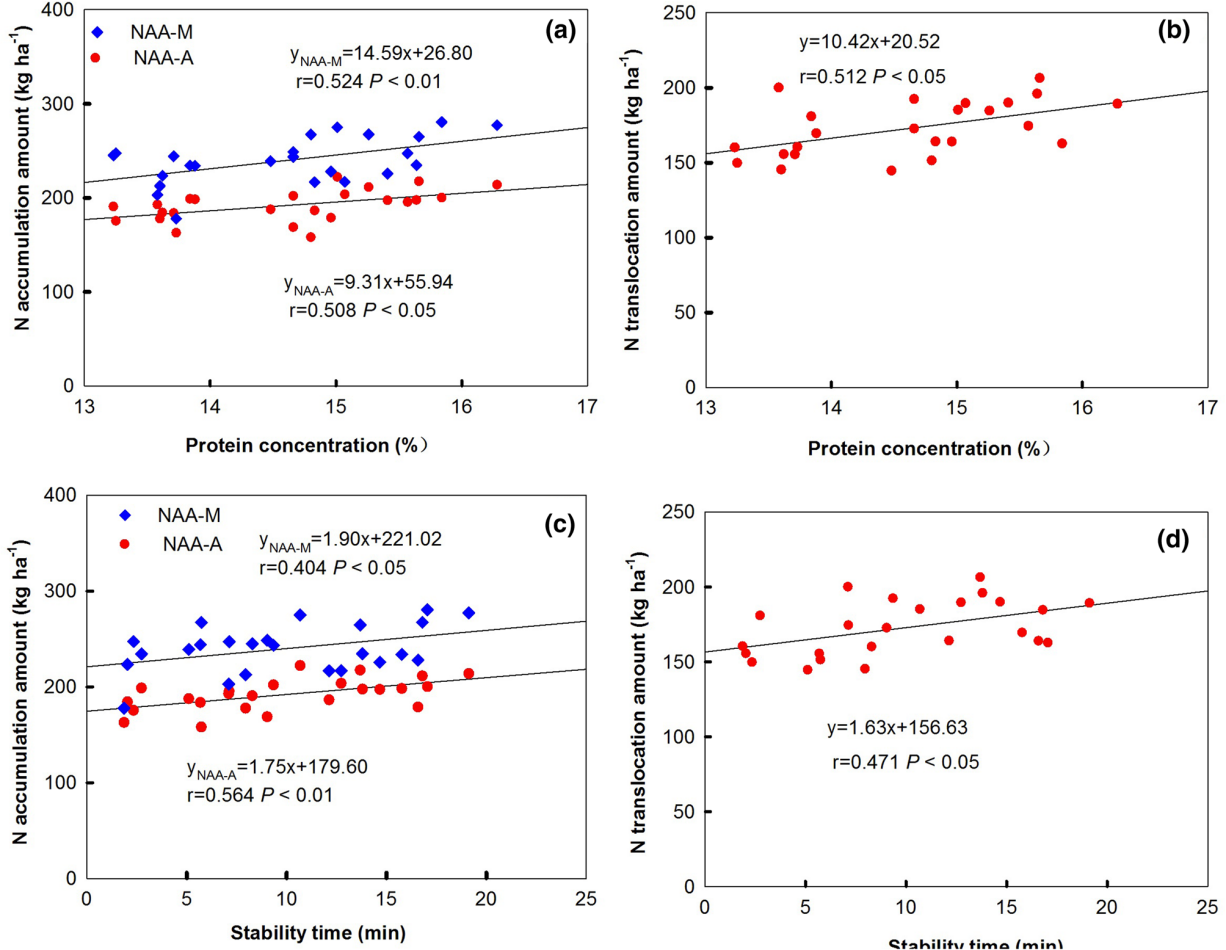
Relationship between N accumulation amount, and protein concentration (**a**), and stability time (**b**), between N translocation amount, and protein concentration (**c**), and stability time (**d**). *NAA-A and NAA-M *N accumulation amount at anthesis and maturity stage, respectively.

**Figure 3:**
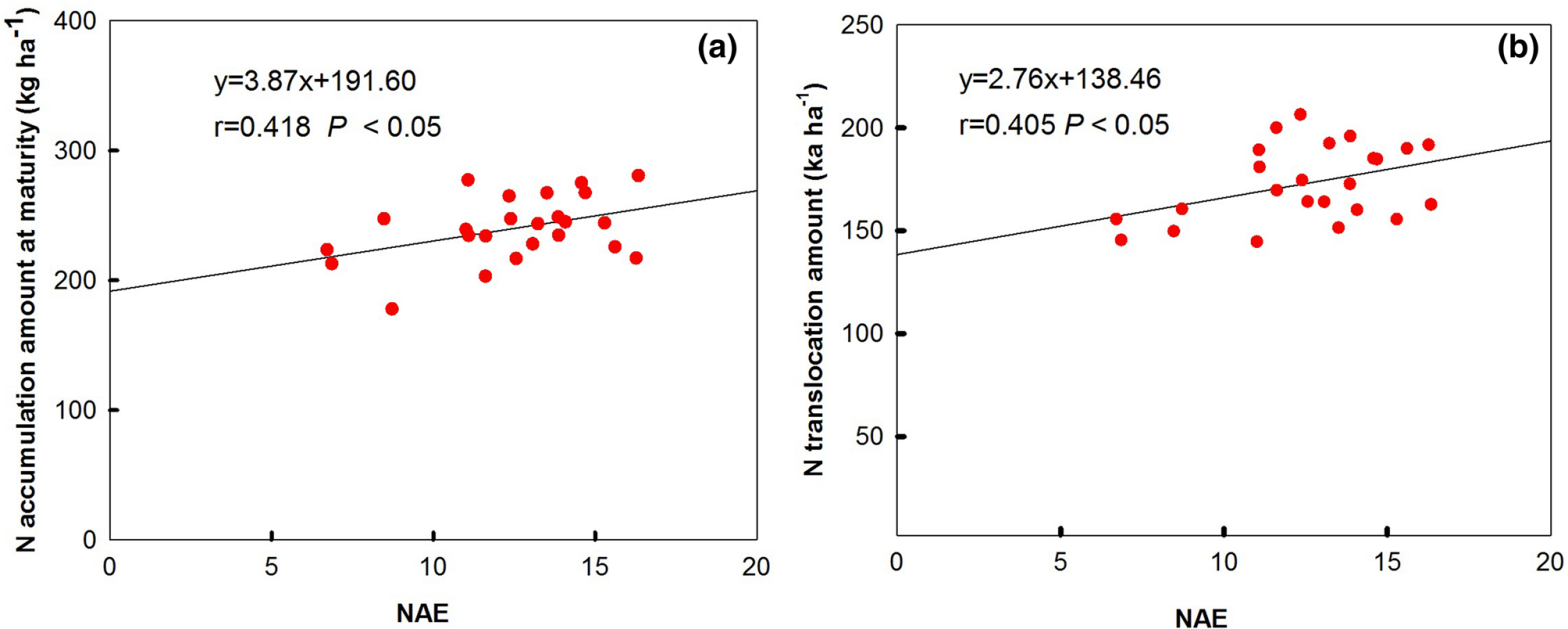
Relationship between NAE and N accumulation amount at maturity stage (**a**), and N translocation amount (**b**). *NAE* nitrogen agricultural efficiency.

As shown in Figure 2, GPC was significantly correlated with NAA-A (r = 0.508, *P* < 0.05), NAA-M (r = 0.524, *P* < 0.01) (Figure 2a), and NTA (r = 0.512, *P* < 0.05) (Figure 2b). Also, DST was significantly and positively correlated with NAA-A (r = 0.564, *P* < 0.01), NAA-M (r = 0.404, *P* < 0.05) (Figure 2c), and NTA (r = 0.471, *P* < 0.05) (Figure 2d). Additionally, NAE was significantly correlated with NAA-M (r = 0.418, *P* < 0.05) (Figure 3a), and NTA (r = 0.405, *P* < 0.05) (Figure 3b). These results indicate that the N translocation amount from vegetative organs to grain play key roles in influencing grain quality and NAE. Similar correlation relationship was also observed between grain quality and physiological traits of wheat cultivars within the same *Glu-D1* allele (Table S1). NTA was positively correlated with GPC and DST in both *Glu-D1* 5 + 10 group and no *Glu-D1* 5 + 10 group. But, the significant correlation was only found within *Glu-D1* 5 + 10 group, with the value being r = 0.740 (*P* < 0.01), and = 0.720 (*P* < 0.01), respectively. Also, NAE was positively significantly correlated with WSC-T, WSCT-P, WSCT-L, and WSCT-T with r = 0.727 (*P* < 0.01), = 0.825 (*P* < 0.01), = 0.629 (*P* < 0.01), and r = 0.764 (*P* < 0.01) within no *Glu-D1* 5 + 10 wheat group, respectively.

## Discussion

Increasing the yield of wheat has always been one of the primary goals of wheat production, especially in a country like China with a large population. With the development of agricultural technology, the yield levels in the HHWWZ increased from 5,500 kg ha^−1^ in 1980s to over 7,500 kg ha^−1^ after 2010^32^. Since the 1980s, the quality of wheat has garnered attention, and some high-quality wheat cultivars (strong gluten wheat) have been bred. However, the quality improvement of these high-quality wheat cultivars is sometimes accompanied by the decrease of GY. The HHWWZ is an irrigated area, and the major wheat production objectives in this zone are to improve wheat yield and the processing quality for pan bread and noodles^24^. Thus, it is very important to achieve a high yield of high-quality wheat in HHWWZ. Here, five wheat cultivars, including ‘Zhengmai 119’, ‘Zhengmai 369’, ‘Fengdencun 5’, ‘Zhoumai 32’, and ‘Sandemai 1’, not only have high GY (the average GY over 7,500 kg ha^−1^), but also have good quality traits (the average GPC, WGC, DST and DWR all meeting the standard of Qualify Classification of Wheat Varieties from the People’s Republic of China^33^). These results indicate that high-quality wheat cultivars could achieve high GY through coordinating C and N metabolism. It is generally considered that HMW-GS 5 + 10 play a great contribution to dough strength^7,30,31^. Here, wheat cultivars ‘Zhengmai 119’, ‘Zhengmai 369’ and ‘Fengdecun 5’ containing the subunit pair 5 + 10 had better grain quality traits; the subunit 5 + 10 may be the basis for their high-quality. However, we also noticed that ‘Zhoumai 32’ and ‘Saidemai 1’ without HMW-GS 5 + 10 also processed high quality traits. These phenomena mainly contributed to other quality related genes, such as low molecular weight glutenin subunits (LMW-GS)^28^. But, we have to mention that wheat cultivars with high-quality subunits (5 + 10) generally have more opportunities to have good grain quality. Especially, we found that cultivars genotyped with 7 + 8 (*Glu-B1*) and 5 + 10 (*Glu-D1*) got a significantly higher GPC, DST, DWR, and NTA than cultivars genotyped with no 5 + 10. As previous reported, selection for subunits/alleles 1, 7 + 8, 5 + 10, and *Glu-A3d* would be more effective for improving gluten quality and pan bread quality^7^.

It is known that the dry matter accumulation or biomass enrichment is the basis for high-yield of grain^34^. In our study, we found that the dry matter accumulation at different growth stages significantly correlated to GY, but the biomass at maturity showed the highest correlation coefficient. Additionally, post-anthesis dry matter accumulation also significantly positive correlated to GY. This suggests that the dry matter accumulation after anthesis than that before anthesis plays a greater role in increased grain-yield. Our results are consistent with the findings of Zhou et al.^35^ and Jiang et al.^36^, who suggested that maintaining high dry matter accumulation after flowering is an effective way to increase wheat yield. WSC in stem and sheath is an important C source for wheat grain and positively correlates to grain yield^17,18^. Xue et al*.*^18^ suggested that much of the WSC in stem during the late grain-filling period can be remobilized to grains. In our study, WSC accumulation and translocation positively correlated to GY, which indicate that increasing stem WSC accumulation during the early grain-filling period and promoting its translocation during the late grain-filling period can potentially increase wheat grain yield. It has been previously reported that different stem internodes respond differently to the environment^37^, and stem WSC in the lower internodes have a greater role in grain yield than the upper internodes under drought^38^. Here, we also found that GY was significantly correlated with WSC accumulation in lower internodes and WSC translocation amount. But, the correlation coefficient between peduncle and GY was slightly higher than that between lower internodes and GY. One possible reason could be that stem was only divided into peduncle and the rest parts of stem in this study. Another reason may be due to the environment conditions wheat cultivar planted. The underlying mechanism of high WSC accumulation and transport may lie in the expression profiles of fructan metabolism related genes; higher expression of genes related to fructan synthesis and degradation at WSC accumulation and translocation stage, respectively^37^. Thus, the close relationship between stem WSC and GY indicate that keeping high WSC content and accumulation could contribute to increasing GY. Slewinski^39^ also pointed out that manipulating the stem WSC is an avenue to stabilize and increase wheat grain, especially in the face of the flourishing population in future. Thus, WSC accumulation could be used as a morphological and physiological criterion for increasing GY in wheat production or high-yield wheat breeding. Moreover, the correlation relationship among GY, grain quality and physiological traits within the same *Glu-D1* allele seem to indicate that there is a relatively high correlation coefficient between nitrogen accumulation and grain quality traits within *Glu-D1* 5 + 10 wheat cultivars. At the same time there is a relatively close correlation between carbon accumulation and transport and grain yield in wheat cultivars without *Glu-D1* 5 + 10 allele. These results further suggest that the coordination of carbon and nitrogen accumulation is very important for high yield and high quality of wheat.

N assimilation and remobilization differ among different wheat cultivars^40^, and the increase in wheat GPC is largely dependent on the accumulation and remobilization of N before flowering^36^. In this study,

N accumulation and translocation significantly positive correlated to GPC and ST, which indicate that the grain processing quality mainly depends on the accumulation and translocation of N, especially the remobilization of N accumulated before anthesis. The genetic factors affecting the relationship of GY-GPC have been explored. It was reported that AD regulated by photoperiod response gene (*Ppd-D1*) affect N uptake between pre- and post-anthsis; photoperiod sensitive alleles (*Ppd-D1b)* are prone to produce higher GY rather than GPC in comparison with insensitive allele (*Ppd-D1a*)^41^. Bogard et al.^42^ suggested that AD may be the genetic factor for post-anthesis leaf senescence which contributes to the negative GPC-GY relationship. In this study, no significant correlation was observed between AD and GY, between AD and GPC. No significant positive correlation coefficients were also observed between GY and GPC, suggesting the possibility of breeding wheat cultivars with high protein contents and high grain yield. Additionally, even there were difference for AD among the test cultivars; all the wheat cultivars possess *Ppd-D1a* allele (Table S2). Further, the HMW-GS 5 + 10 wheat group in this study showed both higher quality traits (especially for GPC) and N accumulation, translocation than no HMW-GS 5 + 10 groups. The 5 + 10 at *Glu-D1* may be one of the internal factors contributing to the GPC by affecting N metabolism. The N metabolism related enzymes activity or genes expression level and regulation factor also contribute to GPC^9,43,44^. Improved NUE would reduce environmental contamination caused by excessive application of N fertilizer, and in turn, increase economic benefit for farmers. Brasier et al.^41^ found that *Ppd-D1* affected NUE, but the difference between insensitive allele (*Ppd-D1b*) and sensitive allele (*Ppd-D1a*) allele varied with N fertilization manner. Here, no significant correlation appeared between NUtE, NAE and AD among these tested wheat cultivars. But, we found that NAE was significantly correlated with N accumulation and translocation amount, which are agreement with the relationship between N translocation and grain quality. The results indicate that increasing N accumulation and translocation would be better to improve grain quality and NAE. In fact, wheat cultivar ‘Fengdecun 5’ got the highest NAE (16.27 kg kg^−1^) and NUtE (37.16 kg kg^−1^), apart from high GY and quality traits. The results indicate that high-yield, good-quality and high-efficiency could be achieved through the selection of high-quality wheat varieties (genetic factor) and coordination of C and N accumulation and translocation.

## Conclusions

Wheat cultivars containing HMW-GS 5 + 10 have more advantages to obtain high grain quality. High-quality gluten subunit gene *Glu-D1* 5 + 10 would be one of the genetic factors contributing to GPC, DST by affecting plant N accumulation and N translocation from vegetative to grain. Stem WSC accumulation and translocation are related to GY, and can be used as s a morphological and physiological selection criterion for increasing GY. The results suggest that good-quality, high-yield, and high-efficiency could be achieved through the selection of high-quality wheat cultivars and coordination of C and N accumulation and translocation.

## Materials and methods

### Experimental design and management

Field experiments were carried out during the wheat-growing season of 2016–2017 (2017) and 2017–2018 (2018) at the experimental sites of Yuanyang (35°05′ N, 113°97′ E) and Xuchang (34°03′ N, 113°85′ E) in the Henan Province of China. The previous crop in all the experimental sites was maize. The soil type was Fluvoaquic soil and the basal fertility values are listed in Supplemental Table S3.The mean temperature and precipitation at the planting sites during the wheat growing season are shown in Supplementary Figure S1. Twenty-four wheat cultivars were selected as representatives of high-quality and high-yield potential among the widely grown cultivars in the HHWWZ; the major wheat-producing region in China that accounts for about 2/3 of China’s wheat production^6^. All the cultivars and their providers are listed in Supplemental Table S4. According to the HMW-GS type, the wheat cultivars were classified into two groups: with HMW-GS 5 + 10, and without HMW-GS 5 + 10. The composition of HMW-GS of these cultivars was listed in Table S2. The experiments were conducted according to a randomized block design with three replicates. The plot size was 3 m (width) × 5 m (length) and the planting density was 250 seed m^-2^. Each plot had 12 rows with equal spacing between the rows. All the plots received 135 kg ha^−1^ of P_2_O_5_ as triple superphosphate and 135 kg ha^−1^ of K_2_O as potassium chloride pre-sowing. 210 kg N ha^−1^ was applied as urea where 50% of the N was applied before sowing and another 50% was applied at the elongation stage. N0 (no N fertilization) treatment for each wheat cultivar with three replicates was used as control to calculate NAE. The growth and development period are listed in Supplementary Table S5. The plants in 9-m^2^ area in each plot were harvested manually when the plants attained physiological maturity. The quality of wheat grains was evaluated after storing them at room temperature for 2 months.

### Measurements

Twenty stems of each plot were sampled at heading, at anthesis, 10 days after anthesis (DAS), and at the maturity stages. At maturity, the plants were separated into leaves, stems with leaf sheaths (these parts were cut into three segments: peduncle and lower internodes, according to Hou et al.^37^), chaff, and grain. The fresh samples were put into an oven at 105 °C for 30 min and then dried at 80 °C until they reached a constant weight for dry weight determination.

### Dry matter translocation

Different parameters related to the dry matter were calculated according to Liu et al.^45^.

Pre-anthesis dry matter translocation (Pre-DT, kg ha^−1^) = dry matter accumulation amount at anthesis − dry matter accumulation amount at maturity (excluding grains).

Post-anthesis dry matter accumulation (Post-DA, kg ha^−1^) = Grains weight at maturity (GW) − Pre-DT.

### Nitrogen accumulation and translocation, nitrogen efficiency

N content of the samples were determined using the Kjeldahl (K1100) procedure, and the accumulation and translocation of N in wheat plants were calculated as follows^22,46^:

- N accumulation amount (kg ha^−1^) = N concentration (%) × dry matter accumulation amount (kg ha^−1^).
- Pre-anthesis N translocation (Pre-NT, kg ha^−1^) = Total aboveground N accumulation amount at anthesis − N of vegetative parts at maturity.
- N agronomic efficiency (NAE) = (Grain yield in N fertilizer treatments − Grain yield in control treatment)/the amount of nitrogen fertilizer applied.
- N utilization efficiency (NUtE) = Grain yield/Above-ground N accumulation at maturity.

### Water-soluble carbohydrates (WSC)

WSC content was determined according to the method of Hou et al.^37^. Briefly, the stem sample (0.10 g) was extracted with 80 °C water for 40 min, centrifuged (4,500 r min^−1^, 20 min), and the supernatant was collected. The extraction process was then repeated twice with 4 ml of 80% ethanol. The WSC content was quantified based on the total sugar content obtained by absorption at 620 nm.

WSC translocation amounts (kg ha^−1^) = WSC accumulation amounts at 10 DAA − WSC accumulation amounts at maturity.

### Flour quality traits

Wheat grains were milled using a laboratory test mill (Brabender Junior) based on an approved method 26-21A (AACC, 1995). The flour protein concentration was determined by a near-infrared transmittance analyzer (Foss Tecator 1241), and the wet gluten content was tested by a gluten testing system (Perten Glutenmatic 2200). The stability time and dough water absorption rates were determined by a Farinograph (Brabender Farinograph-E, Duisburg, Germany) according to an approved method AACC 54–21 (AACC, 1995).

### Identification of HMW-GS and photoperiod genes allele

Glutenin protein extracts were prepared according to He et al*.*^7^, and the fraction was separated by SDS-PAGE analysis according to Singh et al. ^47^. Nomenclature of Payne and Lawrence^48^ was used to classify HMW-GS, and wheat cultivar ‘CS’ with N, 7 + 8, 5 + 10 was used as a reference. The photoperiod genes were determined using gene-specific markers reported by Beales et al.^49^. The HMW-GS composition and photoperiod genes allele were listed in Table S2.

### Statistical analysis

Analysis of variance (ANOVA) was performed using GLM in SPSS 19.0 software (Statistical Program for Social Science) for all traits, with cultivars and environments as fix factors. Differences among cultivars were tested using Tukey’s test. Pearson’s linear correlation analysis was conducted by correlate-bivariate. All the figures were drawn using Origin 9.0 (Origin Lab Corporation, USA).

## Supplementary information

Supplementary file1

Supplementary file2
