# Supplementary figures and images for: Coordination of carbon and nitrogen accumulation and translocation of winter wheat plant to improve grain yield and processing quality
Source: Sci Rep. 2020 Jun 25;10:10340. doi: 10.1038/s41598-020-67343-5 (PMC7316831; doi:10.1038/s41598-020-67343-5)

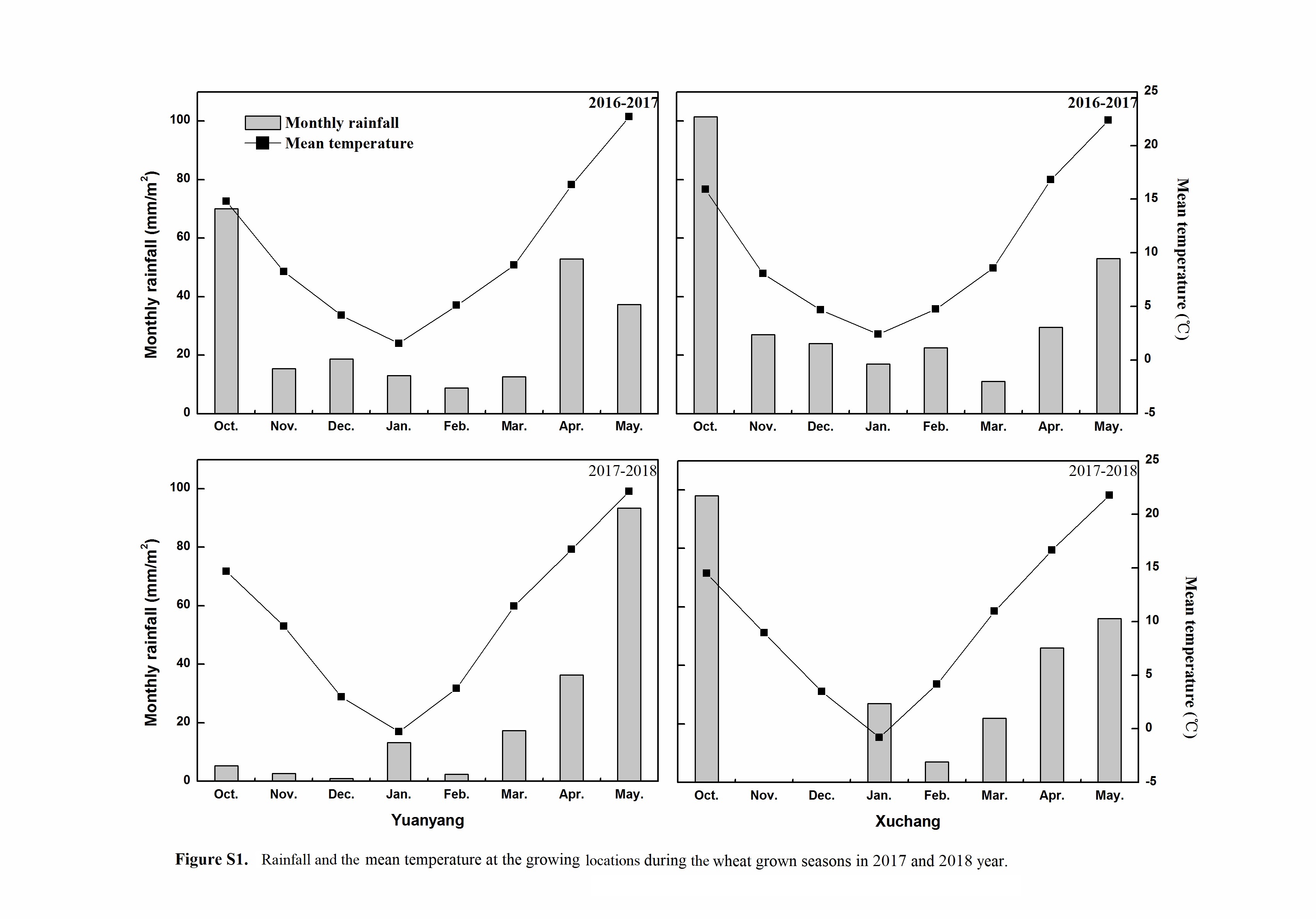

Supplement: Supplementary file 1 — Supplementary file1 [file 41598_2020_67343_MOESM1_ESM.jpg]
