# Supplementary material for: Coordination of carbon and nitrogen accumulation and translocation of winter wheat plant to improve grain yield and processing quality
Source: Sci Rep. 2020 Jun 25;10:10340. doi: 10.1038/s41598-020-67343-5 (PMC7316831; doi:10.1038/s41598-020-67343-5)
Supplement: Supplementary file 2 — Supplementary file2 [file 41598_2020_67343_MOESM2_ESM.docx]

**Supplementary Table S1 Pearson coefficient of correlation among grain yield, quality traits and physiological traits of wheat cultivars within the same *Glu-D1* allele**

| HMW-GS 5+10 |  | GY | KN | TKW | AD | GPC | WGC | DST | DWR | AGBM-J | AGBM-A | WSC-T | WSCT-P | WSCT-L | WSCT-T | LAI-J | LAJ-A | NAA-A | NAA-M | NTA | NAE | NUtE |
| --- | --- | --- | --- | --- | --- | --- | --- | --- | --- | --- | --- | --- | --- | --- | --- | --- | --- | --- | --- | --- | --- | --- |
|  | GY | 1.000 | 0.801** | 0.831** | -0.232 | 0.201 | 0.069 | 0.108 | 0.128 | 0.196 | 0.426 | 0.207 | 0.293 | 0.113 | 0.196 | 0.145 | -0.127 | 0.171 | -0.131 | 0.282 | 0.595* | 0.744** |
| With | GPC | 0.201 | 0.117 | 0.228 | -0.426 | 1.000 | 0.236 | 0.793** | 0.482 | 0.101 | 0.466 | -0.229 | -0.608* | -0.013 | -0.240 | -0.106 | -0.075 | 0.576 | 0.512 | 0.740** | 0.474 | -0.210 |
|  | DST | 0.108 | 0.191 | -0.002 | -0.452 | 0.793** | -0.154 | 1.000 | 0.290 | -0.008 | 0.014 | -0.390 | -0.508 | -0.286 | -0.407 | -0.509 | -0.424 | 0.665* | 0.240 | 0.720** | 0.311 | -0.117 |
|  | NAE | 0.595* | 0.555 | 0.437 | -0.107 | 0.474 | 0.211 | 0.311 | 0.050 | 0.485 | 0.527 | 0.244 | 0.154 | 0.242 | 0.240 | 0.508 | -0.248 | 0.324 | 0.138 | 0.563 | 1.000 | 0.100 |
|  | NUtE | 0.744** | 0.566 | 0.628* | -0.440 | -0.210 | -0.008 | -0.117 | 0.169 | -0.168 | 0.018 | -0.23 | 0.131 | -0.372 | -0.230 | -0.065 | -0.337 | -0.121 | -0.594* | -0.078 | 0.100 | 1.000 |
|  |  | \| GY \| KN \| TKW \| GPC \| WGC \| DST \| DWR \| AGBM-J \| AGBM-A \| WSC-T \| WSCT-P \| WSCT-L \| WSCT-T \| LAI-J \| LAJ-A \| \| --- \| --- \| --- \| --- \| --- \| --- \| --- \| --- \| --- \| --- \| --- \| --- \| --- \| --- \| --- \| | KN | TKW | AD | GPC | WGC | DST | DWR | AGBM-J | AGBM-A | WSC-T | WSCT-P | WSCT-L | WSCT-T | LAI-J | LAJ-A | NAA-A | NAA-M | NTA | NAE | NUtE |
| Without | GY | 1.000 | 0.806** | 0.428 | 0.333 | 0.089 | -0.187 | 0.480 | 0.206 | 0.574 | 0.397 | 0.730** | 0.776** | 0.618* | 0.741** | 0.652* | 0.234 | 0.362 | 0.383 | 0.292 | 0.788** | 0.432 |
|  | GPC | 0.089 | -0.158 | 0.402 | 0.293 | 1.000 | 0.629* | 0.599* | 0.266 | -0.177 | -0.175 | 0.186 | 0.349 | 0.128 | 0.210 | 0.093 | -0.051 | 0.238 | 0.483 | 0.119 | 0.512 | -0.421 |
|  | DST | 0.480 | 0.372 | 0.241 | 0.046 | 0.599* | 0.116 | 1.000 | 0.550 | 0.370 | -0.035 | 0.215 | 0.686* | 0.060 | 0.254 | 0.216 | -0.01 | 0.364 | 0.442 | 0.097 | 0.648* | -0.038 |
|  | NAE | 0.788** | 0.499 | 0.558 | 0.600* | 0.512 | 0.147 | 0.648* | 0.334 | 0.407 | 0.128 | 0.727** | 0.825** | 0.629* | 0.764** | 0.718** | 0.226 | 0.335 | 0.604* | 0.129 | 1.000 | 0.009 |
|  | NUtE | 0.432 | 0.631* | -0.241 | 0.131 | -0.421 | -0.432 | -0.038 | 0.352 | 0.639* | 0.305 | 0.230 | 0.284 | 0.150 | 0.208 | -0.043 | 0.271 | -0.22 | -0.637* | 0.497 | 0.009 | 1.000 |

*, ** Significant at the 0.05 and 0.01 probability level, respectively. GY, Grain yield; KN, Kernels number; TKW, Thousand-kernel weight; GPC, grain protein concentration; WGC, wet gluten content; DST, dough stability time; DWR, dough water absorption rate; AGBM-J, aboveground biomass at jointing stage; AGBM-A, aboveground biomass at anthesis stage; WSC-T, WSC accumulation amount of stem at 10d post-anthesis; WSCT-P, WSC translocation amount of peduncle; WSCT-L, WSC translocation amount of lower internodes; WSCT-T, WSC translocation amount of total stem; LAI-J, leaf area index at jointing stage; LAI-A, leaf area index at anthesis; NAA-A, nitrogen accumulation amount at anthesis; NAA-M, Nitrogen accumulation amount at maturity; NTA, nitrogen translocation amount; NAE, nitrogen agricultural efficiency; NUtE, N utilization efficiency.

**Supplementary Table S2 Composition of HMW-GS and photoperiod gene allele**

| Cultivar | *Glu-A1* | *Glu-B1* | *Glu-D1* | *Ppd-D1* | Cultivar | *Glu-A1* | *Glu-B1* | *Glu-D1* | *Ppd-D1* |
| --- | --- | --- | --- | --- | --- | --- | --- | --- | --- |
| Xinmai 26 | 1 | 7+8 | 5+10 | a | Luomai 26 | 1 | 7+8 | 2+12 | a |
| Zhengmai 366 | 1 | 7+8 | 5+10 | a | Zhoumai 32 | 1 | 7+8 | 2+12 | a |
| Zhengmai 119 | 1 | 7+8 | 5+10 | a | Tianmin 198 | 1 | 14+15 | 2+12 | a |
| Zhengmai 369 | 1 | 7+8 | 5+10 | a | Bainong 4199 | 1 | 7+8 | 2+12 | a |
| Zhengmai 76988 | 1 | 7+8 | 5+10 | a | Zhoumai 27 | 1 | 7+8 | 2+12 | a |
| Fengdecun 5 | 1 | 7+8 | 5+10 | a | Xinhuamai 818 | 1 | 7+9 | 2+12 | a |
| Zhoumia 33 | 1 | 7+8 | 5+10 | a | Xinong 979 | 1 | 7+8 | 2+12 | a |
| Zhoumai 36 | 1 | 7+9 | 5+10 | a | Yunong186 | N | 7+9 | 2+12 | a |
| Luomai 31 | 1 | 7+9 | 5+10 | a | Sandemai 1 | 1 | 7+9 | 2+12 | a |
| Bainong 207 | 1 | 7+9 | 5+10 | a | Shangmai 156 | 1 | 14+15 | 2+12 | a |
| Bainong 307 | 1 | 7+9 | 5+10 | a | Luohan 19 | N | 7+9 | 2+12 | a |
| Yumai 158 | 1 | 7+9 | 5+10 | a | Pingan 11 | N | 14+15 | 2+12 | a |

**Supplementary Table S3**

**Soil physicochemical characteristics of the field experiment in 0-20cm during planting year.**

| Items | Yuanyang |  | Xuchang |  |
| --- | --- | --- | --- | --- |
|  | 2016-2017 | 2017-2018 | 2016-2017 | 2017-2018 |
| Total nitrogen (g kg^-1^) | 0.81 | 0.89 | 1.06 | 0.93 |
| Hydrolyzable nitrogen (mg kg-^1^ ) | 88.94 | 71.64 | 115.50 | 92.71 |
| Organic matter (g kg^-1^) | 10.58 | 12.22 | 16.05 | 15.68 |
| Available phosphorus (mg kg^-1^) | 23.81 | 21.89 | 37.52 | 35.96 |
| Available potassium (mg kg^-1^) | 101.61 | 121.83 | 130.00 | 142.12 |
| pH | 7.92 | 7.90 | 8.08 | 7.94 |
| Texture | Loamy | Loamy | Loamy | Loamy |

**Supplementary Table S4 Names of wheat cultivar in this study and seed provider**

| Cultivar | Provider | Cultivar | Provider |
| --- | --- | --- | --- |
| Bainong 207 | Henan Institute of Science and Technology | Luomai 26 | Luoyang Academy of Agricultural and Forestry Sciences |
| Bainong 307 | Henan Institute of Science and Technology | Luomai 31 | Luoyang Academy of Agricultural and Forestry |
| Yunong 186 | Henan Agricultural University | Zhoumai 32 | Zhoukou Academy of Agricultural Sciences |
| Tianmin 198 | Henan Tianmin Seed Company | Zhoumai 36 | Zhoukou Academy of Agricultural Sciences |
| Xinmai 26 | Xinxiang Academy of Agricultural Sciences Sciences  河南省新乡市农业科学院 | Zhoumia 33 | Zhoukou Academy of Agricultural Sciences |
| Bainong 4199 | Henan Institute of Science and Technology | Xinhuamai 818 | Henan Agricultural University |
| Zhengmai 366 | Henan Academy of Agricultural Sciences | Xinong 979 | Northwest Agricultural and Forestry University |
| Zhengmai 379 | Henan Academy of Agricultural Sciences | Fengdecun 5 | Tiancun Seed Company |
| Zhengmai 119 | Henan Academy of Agricultural Sciences | Sandemai 1 | Sande Seed Company |
| Zhengmai 369 | Henan Academy of Agricultural Sciences | Shangmai 156 | Shangqiu Academy of Agricultural and Forestry Sciences |
| Zhengmai 76988 | Henan Academy of Agricultural Sciences | Luohan 19 | Luoyang Academy of Agricultural and Forestry |
| Yumai 158 | Luohe Academy of Agricultural Sciences | Pingan 11 | Pingan Seed Company |

**Supplementary Table S5 Developmental stages of wheat in 2017 and 2018 planting year**

| Site | Sowing date | Jointing date | Heading date | Anthesis | Maturity |
| --- | --- | --- | --- | --- | --- |
| 2016-2017 | |  |  |  |  |
| Yuanyang | 15 October | 15 March~26 March | 11 April~19 April | 23 April~29 April | 30 May~2 June |
| Xuchang | 30 October | 14 March~20 March | 14 April~19 April | 21 April~27 April | 25 May~29 May |
| 2017-2018 |  |  |  |  |  |
| Yuanyang | 14 October | 14 March~25 March | 10 April~20 April | 25 April~30 April | 30 May~1 June |
| Xuchang | 28 October | 12 March~18 March | 11 April~17 April | 19 April~24 April | 25 May~31 May |
